# Hyperspectral Leaf Image-Based Cucumber Disease Recognition Using the Extended Collaborative Representation Model

**DOI:** 10.3390/s20144045

**Published:** 2020-07-21

**Authors:** Yuhua Li, Zhihui Luo, Fengjie Wang, Yingxu Wang

**Affiliations:** College of Engineering, Nanjing Agricultural University, Nanjing 210031, China; 32316420@njau.edu.cn (Z.L.); 2019212011@njau.edu.cn (F.W.); 2017112018@njau.edu.cn (Y.W.)

**Keywords:** cucumber disease recognition, hyperspectral imaging, extended collaborative representation (ECR), spectral library

## Abstract

Collaborative representation (CR)-based classification has been successfully applied to plant disease recognition in cases with sufficient training samples of each disease. However, collecting enough training samples is usually time consuming and labor-intensive. Moreover, influenced by the non-ideal measurement environment, samples may be corrupted by variables introduced by bad illumination and occlusions of adjacent leaves. Consequently, an extended collaborative representation (ECR)-based classification model is presented in this paper. Then, it is applied to cucumber leaf disease recognition, which constructs a pure spectral library consisting of several representative samples for each disease and designs a universal variation spectral library that deals with linear variables superimposed on samples. Thus, each query sample is encoded as a linear combination of atoms from these two spectral libraries and disease identity is determined by the disease of minimal reconstruction residuals. Experiments are conducted on spectral curves extracted from normal leaves and the disease lesions of leaves infected with cucumber anthracnose and brown spot. The diagnostic accuracy is higher than 94.7% and the average online diagnosis time is short, about 1 to 1.3 ms. The results indicate that the ECR-based classification model is feasible in the fast and accurate diagnosis of cucumber leaf diseases.

## 1. Introduction

Plant diseases severely threaten the yield and quality of agricultural products. Rapid, accurate, and reliable disease detection and identification is vital to disease prevention and control for sustainable agriculture and food security [1]. Traditional methods rely on agronomists manually checking the plant disease symptoms or visible signs of a pathogen with the naked eye [2,3] or professional analysts performing physiological and biochemical analysis including molecular, serological, and deoxyribose nucleic acid [4,5]. Meanwhile, the visual assessment method requires plant to show visible symptoms, which is often used in the middle to late stage of infection [2]; besides, the diagnostic result is heavily influenced by the subjective consciousness and empirical knowledge of observers. As for the method of physiological and biochemical analysis, it is time-consuming and labor-intensive [6], and specific operating environment as well as high level of expertise and operating skills of the analyst are highly demanded to obtain reliable diagnosis results. 

With the rapid development of computer vision and artificial intelligence, image processing techniques have shown great potential in automatic disease diagnosis, which can overcome some defects of the above methods and mitigate the problem of lack of expertise in the field of agriculture [7]. By now, numerous image processing-based diagnosis methods or systems have been developed by researchers and have achieved great success [1,8,9,10,11,12,13]. For instance, based on image processing techniques and artificial neural networks, Pawar et al. [1] proposed a real-time cucumber disease detection system that consisted of five sequential procedures, including image acquisition, preprocessing, feature extraction, creating database and classification, providing classification accuracy of 80.45% on cucumber downy mildew, powdery mildew, and healthy plants. Zhang et al. [9] segmented diseased blade images by the K-means clustering method, extracted the shape and color features from the lesions, and utilized the sparse representation classifier to achieve rapid identification of cucumber diseases. Based on leaf images, Sladojevic et al. [10] utilized deep convolutional neural networks to distinguish 13 different types of diseases out of healthy leaves and achieved precision between 91% and 98%. In reference [11], Ferentinos trained several convolutional neural network models using a large open database containing of 58 classes, and realized disease diagnosis using simple blade images from healthy and diseased plants. Jia et al. [12] segmented blade images by the edge detection method and OTSU method to extract the diseased areas, and used neural networks to improve the recognition rate of cucumber bacterial angular spot and downy mildew diseases. Singh [13] proposed a sunflower leaf disease detection method using image segmentation based on particle swarm optimization and achieved average classification accuracy of 98% on the visible light leaf images of six diseases. From the above described methods, it can be seen that image processing technique-based methods basically rely on extracting manifold features like the color, dispersion, texture, shape, gray levels, and connectivity from the lesions in visible light blade images [3], and then, train classification models or directly utilize the existing classifiers to identify the type of disease. However, there are no guidelines of feature selection to decide which features are better that can be used [14]. Moreover, in the early stage of infection, disease symptoms are often unobvious or even asymptomatic, causing visible light image-based methods to hardly be used for disease early diagnosis. 

The hyperspectral imaging (HSI) technique simultaneously obtains information at the two-dimensional spatial image level and spectrum level with wavelengths from 400 to 2500 nm, which not only can reflect the plant surface changes but also the inner physiology and composition changes [15] caused by biotic plant stresses, such as diseases, pests, and weeds. Thus, it has been increasingly used for plant disease diagnosis, even in the cases of unobvious or invisible symptoms [6] during recent decades. For instance, López-López et al. [16] calculated the canopy temperature and vegetation indices from the high-resolution hyperspectral and thermal imagery of almond, and then, applied linear discriminant analysis and support vector machine (SVM) methods to their combination to distinguish the severity levels of red leaf blotch. Rumpf et al. [17] used SVM and spectral vegetation indices to early detect sugar beet diseases, achieving classification accuracies up to 97% for the discrimination between healthy sugar beet leaves and diseased ones. Yuan et al. [18] conducted autocorrelation analysis on spectral features, disease-sensitive bands, and new disease indices to generate an optimized spectral feature set, and based on which, tea anthracnose detection is realized by developing a framework combining unsupervised classification and adaptive two-dimensional thresholding. Gao et al. [19] used the sequential feature selection algorithm to select the spectral feature wavelengths and utilized neural networks to classify the early ripeness of strawberry based on the selected spatial feature images. Tian et al. [20] extracted the chromaticity moments-based texture features of the filtered diseased leaf images in several characteristic wavelengths and used SVM to classify cucumber downy mildew and powdery mildew.

By analyzing the existing visible and HSI-based methods, it can be found that machine learning techniques (such as SVM, artificial neural network, ensemble learning, etc.) have been more and more widely used in disease diagnosis and obtain superior performance than traditional means. They establish or learn models from the empirical data using computers and when faced with new situation or data, the learned models will give the corresponding judgement [21]. For classification tasks like image classification, face recognition, as well as plant disease identification, it is commonly believed that machine learning techniques require sufficient training data per subject that can span the variations of testing samples. However, acquiring appropriate and useful agricultural data is usually laborious and time-consuming [22]; only a few training samples per subject can be offered in many practical cases, thus, the trained models derived from insufficient training samples lack good ability of generalization and hence, may be unsuitable for testing samples with unsatisfactory performances. For this problem, an ECR-based classification model is presented and then, cucumber disease recognition is used as a proof-of-concept. In more detail, we extract the spectral curves of the pixels in the lesions of diseased leaves as samples. Since each sample can be regarded as a superposition of a pure spectral curve and a linear variable introduced by illumination and occlusions (this paper mainly considers the common linear variations), we cast them as a linear combination of atoms from a pure spectral library and a variation spectral library, respectively. The former spectral library is utilized to distinguish from different types of diseases based on the corresponding collaborative representation coefficients, while the latter alleviates the influence of the linear variations. 

The rest of this paper is organized as follows. Section 2 introduces the Materials and Methods. More specifically, Section 2.1 describes how the inoculation experiment is conducted; Section 2.2 describes the acquisition and preprocessing of hyperspectral images; Section 2.3 briefly reviews the related collaborative representation (CR)-based classification model [23]; by extending CR to ECR, an ECR-based classification model is presented in Section 2.4; Section 2.5 uses cucumber disease recognition as a proof-of-concept for the ECR-based classification model; Section 2.6 briefly introduces the experiment setup. The experimental results and analyses of cucumber disease recognition are given in Section 3. Section 4 concludes the paper. 

## 2. Materials and Methods

### 2.1. Inoculation Experiment

In this study, a vigorous cucumber variety in China named ‘Lufeng’ was used as the object. We purchased the cucumber seeds from a commercial seed store located near Jiangsu Academy of Agricultural Sciences in Nanjing (China). The experiment was conducted between March and May 2019. We cultivated about 120 cucumber plants in our greenhouse; when they grew to have three real leaves, 55 healthy cucumber plants with similar growth state were selected for experiments. Among these, 5 plants were randomly selected and constituted the healthy control group (group A). The rest formed the inoculation group, in which, 25 plants were inoculated with Corynespora cassiicola (group B), while the remaining 25 plants were inoculated with anthracnose (group C). For each plant, 2 real leaves were inoculated. The strains of Corynespora cassiicola and anthracnose used in experiments were purchased from the Agricultural Culture Collection of China. The inoculation was carried out by artificially making small wounds on leaves using a sharp blade and then, covering the wounds with small mycelia blocks. To avoid cross-infection, plants in group A, B, and C were separately placed in different artificial environment boxes of the same type, with the relative humidity being kept as 90%, while two temperatures (28 °C for 16 h and 24 °C for 8 h) alternated with each other. 

### 2.2. Hyperspectral Image Acquisition and Preprocessing

**Figure 1:**
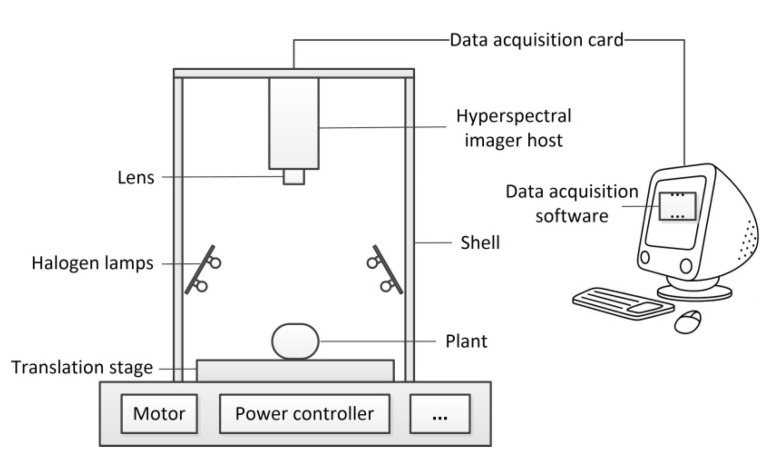
Schematic diagram of the hyperspectral image acquisition system.

About 24 h after inoculation, the hyperspectral images of any two leaves of each plant in group A and all the inoculated leaves in group B and C were acquired every 24 h using an indoor push-broom HSI system GaiaSorter (Dualix spectral imaging, Chengdu, China), which was composed of two imaging units (391–1045 and 1000–2500 nm), a horizontal electronically controlled translation stage (HSIA-T1000), an image acquisition software (SpecView), eight halogen lamps (HSIA-LS-T-H) with 400 W, and an equipment shell made of a steel plate [5]. A schematic diagram of the hyperspectral image acquisition system is shown in Figure 1. Here, we only collected the raw hyperspectral images corresponding to 391–1045 nm because the acquisition of hyperspectral images corresponding to 1000–2500 nm would take a relatively longer time and our manpower was very insufficient. The lens was about 25 cm above the leaf surface and the exposure time was set as 7.5 ms. The spatial and spectral resolution of the collected raw hyperspectral image was 1394 × 1024 pixels and 2.8 nm, respectively. 

Affected by the uneven distribution of light intensity, the dark current of the sensors, and atmospheric turbulence, the raw hyperspectral images contained some noise, which were eliminated through black and white correction in Equation (1):

(1) $$I=\frac{I_{raw}-I_{dark}}{I_{white}-I_{dark}}\times 100\%$$

where $I_{raw}$ and I were the raw and corrected hyperspectral images, respectively; $I_{dark}$ was the dark calibration image obtained by covering the lens cap (0% reflectivity); $I_{white}$ was the white calibration image obtained by scanning the standard Teflon white board (99% reflectivity). It is worth noting that if our data are used for comparisons with other research, a further correction with formula of I×99% is needed. The corrected hyperspectral images were used for further analysis. In the groups inoculated with diseases, if one leaf had no visible signs of infection, we ignored it and did not extract any pixels from it. For the infected plants in group B, we avoided the pixels near the edges of disease spots and randomly extracted 4000 spectral curves of pixels inside the disease spot by hand. The same operation was performed on group C. As for the healthy plants in group A, 4000 spectral curves of pixels were manually and randomly extracted from the corrected hyperspectral images. The basic information of the groups was briefly described in Table 1. Each spectral curve owned 256 elements corresponding to the 256 wavebands from 391–1045 nm, with an interval of 2.8 nm. Unless otherwise stated, for each group, 10 spectral curves were randomly selected from the well extracted 4000 spectral curves for training the recognition model, and the remaining were used for testing and verifying. Then, each spectral curve was vectorized to a 256-dimensional column vector and considered as a sample. In total, there were three types of treatments with 30 training samples that constitute a training set and 11,970 testing samples that constitute a testing set. For each experiment below, the training and testing sets are regenerated in the above manner each time. 

To preliminarily reduce the adverse impact of irrelevant information while retaining effective sample information for the subsequent disease recognition as much as possible [24], we first preprocessed the spectral curve samples. Since preprocessing methods often have a significant influence on disease recognition results and different methods have their own types of interference that they are good at dealing with, for example, Savitzky-Golay convolution smoothing (SG) can effectively remove random noise while preserving image details and profile information; multiplicative scatter correction (MSC) can handle the problem of scattering effect; derivative spectrum can eliminate baseline and other background interference [25], and etc. Given that, one should choose an appropriate method according to the specific situation [24] and possible sources of noise generated in the acquisition process. Among the commonly used spectral preprocessing methods, the effects of SG, MSC, moving average smoothing (MAS), second derivative computed by SG, and standard normal variate (SNV) are evaluated in Section 3 and the related parameters of the above methods are listed in Table 2. After preprocessing, the principal component analysis (PCA) method was utilized to reduce the dimensionality of spectral curve samples to cut down the subsequent calculation and time costs. Besides, the reflectance of different wavelengths may contain some redundant information and PCA can get rid of the redundancy and retain sample information as much as possible by increasing the sampling density. The dimension-reduced samples were used for further analysis. 

### 2.3. The Related Work—CR-Based Classification Model

Since ECR is a simple extension of CR, here, we first briefly introduce the CR-based classification model [23]. Denote all the training samples of k diseases as the matrix $Y=\left[Y_{1},Y_{2},\cdots ,Y_{k}\right]\in R^{d\times n}$, where the submatrix $Y_{i}\in R^{d\times n_{i}}$ stacks the $n_{i}$ training samples of the ith class of disease, d is the length of each sample, and n is the total number of training samples. Assuming that y is a a testing sample, it is firstly collaboratively represented as the linear combination of all the columns of Y, as follows:

(2) y=Ya+ϵ

where a is the CR coefficient and ϵ is the reconstruction error. Then, the testing sample y will be classified to the class which leads to a minimum reconstruction error:

(3) $$\underset{j}{j^{*}=argmin}||y-Y\delta_{j}(a){\left|\right|}_{2}^{2}$$

where $\delta_{j}\left(a\right)$ is obtained by preserving the coefficients corresponding to the jth class and set the rest coefficients to zeros; $j^{*}$ is the obtained class label of the testing sample y. 

Though the CR-based classification method is a smart and excellent classification model [26,27,28], the premise of its success is that there are enough training samples for each class, otherwise it may fail to achieve high classification accuracies [29]. However, in practical scenarios, there may be insufficient training samples for each class due to the limitation of manpower, time, and collection environment. Under these circumstances, the CR-based classification model may not be able to span the variations of testing samples and thus could not ensure good classification performance.

### 2.4. The ECR-Based Classification Model

To overcome the drawback of the CR-based classification model and inspired by the studies in references [30] and [31], this paper presents an ECR-based classification model. It aims at a reduction in the required training sample for each class and an alleviation of the adverse impact of linear interferences in samples. The ECR-based classification model is composed of two sequential procedures: an offline stage that offline constructs two dictionaries and an online recognition stage that determines the identity of the testing sample, which are respectively described in detail as follows (in Section 2.4, the symbols and meanings of variables are the same as in Section 2.3).

#### 2.4.1. The Offline Preparation of Dictionaries

Assuming each sample is a superimposition of a pure sample and a disturbing variable, a pure dictionary $D_{P}$ and a variation dictionary $D_{V}$ are firstly created to well collaboratively represent the former and the latter, respectively. Let the mean vector of $Y_{i}$ be $c_{i}=\frac{1}{n_{i}}Y_{i}h_{i}$, where $h_{i}$ is a $n_{i}$-dimensional column vector of all ones. The pure dictionary $D_{P}$ and the variation dictionary $D_{V}$ are offline constructed according to the same method from Gao et al. [31]:

(4) $$D_{P}=\left[c_{1},c_{2},\cdots ,c_{k}\right]\in R^{d\times k}$$

(5) $$D_{V}=\left[Y_{1}-c_{1}h_{1}^{T},\cdots ,Y_{k}-c_{k}h_{k}^{T}\right]\in R^{d\times n}$$

These two dictionaries are stored in a computer for recall in the subsequent online recognition stage, which is described below.

#### 2.4.2. The Online Recognition Stage

Given a testing sample y of unknown identity, it is firstly decomposed as a linear combination of atoms from dictionaries $D_{P}$ and $D_{V}$, based on the ECR model, as follows:

(6) $$y=y_{P}+y_{V}+\varepsilon =D_{P}\alpha +D_{V}\beta +\varepsilon$$

where $y_{P}\approx D_{P}\alpha$ represents the pure sample component of y, $y_{V}\approx D_{V}\beta$ denotes the disturbing variable superposed on $y_{P}$, ε is a small reconstruction error term. $\alpha =\left[\alpha_{1},\alpha_{2},\cdots ,\alpha_{k}\right]\in R^{k\times 1}$ and $\beta =\left[\beta_{1},\beta_{2},\cdots ,\beta_{n}\right]\in R^{n\times 1}$ are the ECR coefficient vectors corresponding to $D_{P}$ and $D_{V}$, respectively. They can be easily obtained by solving the following $l_{2}$-norm regularized least square problem:

(7) $$\left[\begin{array}{c} \hat{\alpha }\\ \hat{\beta } \end{array}\right]=\underset{\alpha ,\beta }{argmin}\left\{\|y-\left[D_{P},D_{V}\right]\left[\begin{array}{c} \alpha \\ \beta \end{array}\right]{\|}_{2}^{2}+\mu \|{\left[\begin{array}{c} \alpha \\ \beta \end{array}\right]\|}_{2}^{2}\right\}$$

where μ is a manually tuned parameter that balances the reconstruction fidelity term and the regularization term. The solutions $\hat{\alpha }$ and $\hat{\beta }$ are the estimations of α and β, and they can be analytically derived as:

(8) $$\left[\begin{array}{c} \hat{\alpha }\\ \hat{\beta } \end{array}\right]={\left(D^{T}D+\mu I\right)}^{-1}D^{T}y$$

where $D=\left[D_{P},D_{V}\right]$ is the cascade of $D_{P}$ and $D_{V}$. Based on the ECR coefficient vector ${\left[{\hat{\alpha }}^{T},{\hat{\beta }}^{T}\right]}^{T}$, the identity $i^{*}$ of the testing sample y is determined by evaluating which class results in the minimum reconstruction residual, as follows:

(9) $$\underset{i}{i^{*}=argmin}||y-\left[D_{P},D_{V}\right]{\left[\begin{array}{c} \delta_{i}\left(\hat{\alpha }\right)\\ \hat{\beta } \end{array}\right]||}_{2}^{2}$$

where $\delta_{i}\left(\hat{\alpha }\right)$ is a column vector obtained by preserving the coefficients of α corresponding to the ith class and setting the remaining to zeros. 

To show the ECR-based classification model more concisely, the detailed steps are summarized as Algorithm 1.

**Algorithm 1.** The ECR-based classification model**Input**: the testing sample y, the training samples $Y=\left[Y_{1},Y_{2},\cdots ,Y_{k}\right]\in R^{d\times n}$, parameter μ. **Output**: the identity $i^{*}$ of the testing sample y. The offline preparation of dictionaries: 1: Construct pure dictionary $D_{P}$ and variation dictionary $D_{V}$ using Equations (4) and (5), respectively. 2: Store dictionaries $D_{P}$ and $D_{V}$ in a computer for recall. The online recognition: 1: Represent y as $y=D_{P}\alpha +D_{V}\beta +\varepsilon$ and solve ECR coefficient vectors α and β using Equation (8). 2: Determine the identity of y by Equation (9).

### 2.5. Cucumber Leaf Disease Recognition Using the ECR-Based Classification Model

**Figure 2:**
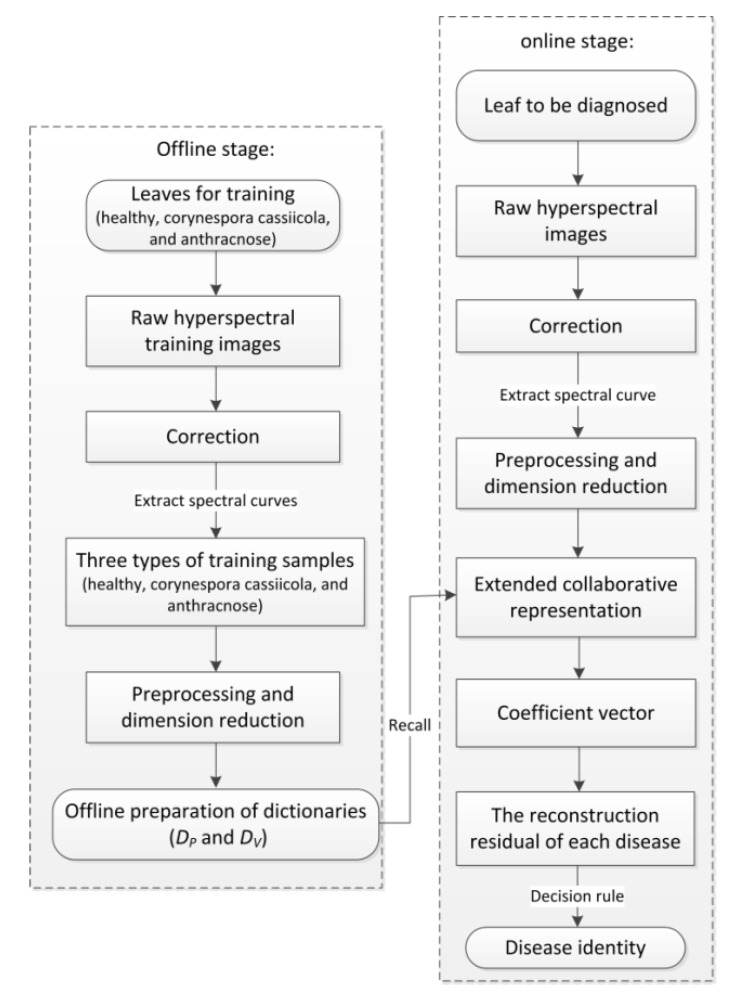
The flowchart for cucumber disease recognition using the ECR-based classification model.

As a proof-of-concept, we apply the ECR-based classification model to cucumber leaf disease recognition in this section. In practical cucumber production, collecting suitable and useful disease data is time-consuming, labor-exhaustive, and controlled environment-demanding. Moreover, the acquisition environment such as illumination condition and occlusions from adjacent leaves may introduce linear variations and then, overlay the pure spectral curves of pixels in hyperspectral leaf images. As a result, each spectral curve sample obtained in Section 2.2 can be regarded as a superimposition of a pure spectral curve and a linear variation. Due to the above two reasons, there may be insufficient training spectral curve samples per type of disease. Under this circumstance, we try to verify the feasibility and effectiveness of ECR-based classification model on cucumber leaf disease recognition. Firstly, the pure dictionary $D_{P}$ and the variation dictionary $D_{V}$ are constructed base on Equations (4) and (5) using the training spectral curve samples (here, “dictionary” can also be called “spectral library”). Then, they are used to collaboratively represent the pure spectral curves and the linear variations, respectively. By doing this, not only the adverse impact of linear interferences but also the requirement for the number of training samples can be reduced. Given any query spectral curve sample whose disease type is unknown, it should be firstly decomposed using the spectral libraries $D_{P}$ and $D_{V}$. Then, the ECR coefficient vector is calculated by solving a $l_{2}$-norm regularized least square problem (7), and afterwards, is utilized to identify the disease type by evaluating which disease leads to the minimum reconstruction residual in terms of Equation (9). The detailed description of cucumber disease recognition using the ECR-based classification model is summarized in Figure 2, which is divided into two parts: an offline stage for the preparation of two spectral libraries and an online stage for cucumber disease recognition. 

### 2.6. Parameter Settings

To assess the performance of the ECR-based classification model on cucumber disease recognition, several experiments were conducted on the hyperspectral images of healthy leaves and leaves infected with anthracnose and Corynespora cassiicola in Section 3. We also compared with the performances of five other commonly used classic classifiers or recognition methods: SVM, K-means clustering (K-means), and linear discriminant analysis classifier (LDA), random forests (RF), and the extended sparse representation classifier (ESRC) [30]. According to experimental experiences, the regularization parameter μ in the ECR and ESRC methods is set as 0.001; the number of decision trees in RF is set as 200; the kernel function used in SVM is a radial basis kernel function defined by:

(10) $$K\left(x,y\right)=\exp\left(-\frac{||x-y{\left|\right|}^{2}}{2\sigma^{2}}\right)$$

where $\sigma^{2}$ is set as 1/3; the cluster number in K-means method is set as 3 according to the number of types of disease. 

## 3. Results and Discussion

### 3.1. Effects of Different Preprocessing Methods

**Figure 3:**
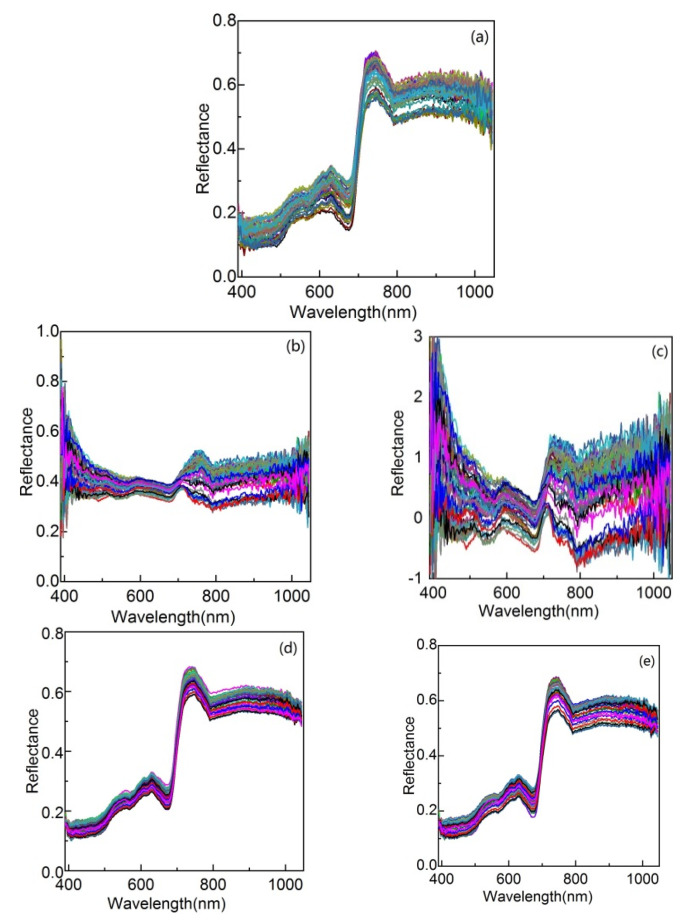
Spectral curve samples preprocessed by different methods. (**a**) Example of the spectral curve samples of anthracnose; (**b**) MSC; (**c**) SNV; (**d**) MAS; (**e**) SG.

In this section, we first preprocess the spectral curve samples by different preprocessing methods to improve the signal-to-noise ratio, then, evaluate their effects on the ultimate disease recognition accuracy of different recognition methods, and finally, based on which, find the one that relatively fits our data. We take anthracnose as an example and randomly choose about 50 spectral curves of anthracnose disease from the training and testing sets, and the prepressing results by different methods are shown in Figure 3. It can be seen from Figure 3a that due to the absorption of radiant energy, two absorption valleys appear near 450 and 670 nm, while there is a reflection peak between them [15]; the signal-to-noise ratio at the beginning and end of the spectral curves is obviously low; although different spectral curves of the same disease are quite different, their shapes are very similar [32]. Figure 3b,e show that SG and MA can effectively smooth the glitch noise in the spectral curve [33]; compared with MSC and SNV, the preprocessed samples of MA and SG contain less noise and are more concentrated with similar shape and appearance. Hence, it is reasonable to believe that the noise contained in our data are mainly random noise. The cucumber disease recognition accuracies of different recognition methods using the preprocessed samples are shown in Table 3. The following phenomenon can be seen: different preprocessing methods have different effects on the same recognition method; MAS and SG can lead to higher disease recognition accuracies, regardless of the recognition methods and the reason may be that these smoothing methods are very suitable for dealing with the type of noise in our data, making the variations between samples of the same disease smaller. In contrast with SG, MAS performs better in most cases; the relatively optimal preprocessing method of SVM, LDA, and ECR is MAS, while that of the K-means is SG. Based on the results in Figure 3 and Table 3, we choose MAS to preprocess spectral curve samples. Since the window width of MAS can have a profound effect on classification success, we test the disease recognition accuracies of the presented ECR-based disease recognition method when different window widths are adopted. The results are shown in Table 4, based on which, we identify a relatively better window width 7 for our data in the subsequent experiments. 

### 3.2. Effects of the Variation Spectral Library and the Number of Principal Components

**Figure 4:**
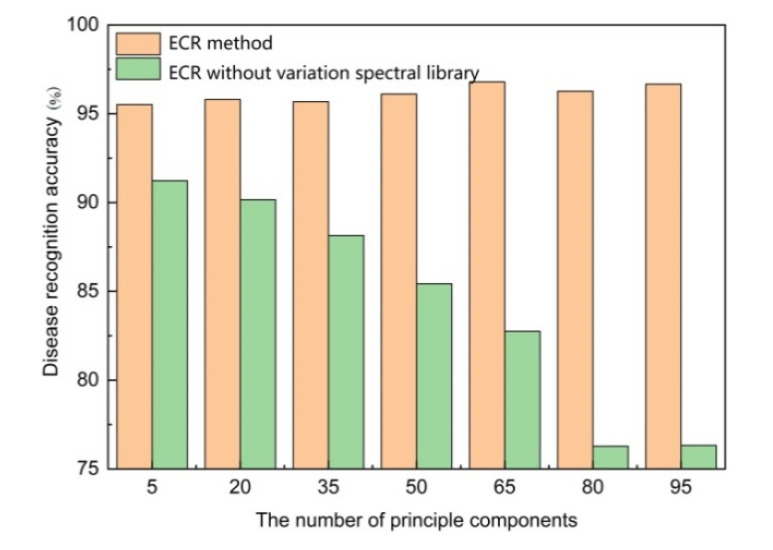
Comparison results of the ECR method with and without variation spectral library.

In order to reduce the calculation and time costs of the subsequent processing, a mainstream dimensionality reduction method named PCA is used to project the preprocessed samples into a low-dimensional space, where the first few principal components contain the most useful information of the sample. The number of principal components retained is an important parameter usually specified by the user in advance. A too small number will cause the loss of much useful information, while a large number will result in the inability to eliminate redundant information. Both the above two cases will cause a negative impact on disease recognition. Here, an experiment is conducted to evaluate the effects of the number of principal components on the ultimate disease recognition accuracy and the results are shown in Table 5. It can be seen that compared with SVM and LDA, ESRC, ECR, and K-means are relatively insensitive and robust to the number of principal components to a certain extent and ECR achieves the highest recognition accuracies, while the performance of ESRC and K-means is comparable. For the supervised machine learning methods SVM and LDA, their recognition accuracies decrease with the increase in the number of principal components; the reason may be that in the case of a small number of training samples, more principal components are selected, and overfitting is more likely to occur and result in a sharp decline in the generalization capability of the recognition model learned from the training set. As for the unsupervised classification method K-means, it has a stable recognition rate, under the condition that the cluster number is set as the groundtruth number of disease type; whereas, if the cluster number is not set beforehand, the recognition accuracy varies, especially when the number of principal components is greater than 80, and the recognition rate falls below 63.89%. As introduced in Section 2, a variation spectral library is constructed to eliminate linear interferences superimposed on the ideal pure spectral curve samples, which may influence the final disease recognition results. Here, we carry out an experiment to verify its effectiveness by separately executing ECR with and without the variation spectral library and the results are shown in Figure 4; it can be seen that the variation spectral library can promote disease recognition accuracy.

### 3.3. Disease Recognition Using Different Methods

The success of the ECR-based classification depends on each disease having sufficient training samples that can span the variations of query samples. However, the premise is not always satisfied due to the variable collection environment and a great consumption of manpower and time. Here, an experiment is carried out to assess the influence of enrollment size (the number of training samples per type of disease) on the performance of the ECR method. The training and testing sets are prepared as follows: for each type of disease, *m* samples are randomly selected from the 4000 extracted spectral curve samples and the rest are used for testing; thus, there are, in total, 3*m* training samples and 12,000 3*m* testing samples. Table 6 shows the disease recognition accuracies of ECR, ESRC, SVM, LDA, and K-means when different enrollment size *m* is adopted, where *m* varies from 20 to 100 with an interval of 10. It can be seen that the ECR method achieves the highest and relatively stable recognition accuracy, with a maximal value of 98.5%. In most cases, ESRC ranks second with a maximal value of 95.5%, whereas the recognition rate of the ESRC method falls below 92%, when enrollment size is smaller than 10. The performances of SVM and LDA have greatly improved as the enrollment size increased, implying that the models learning from a large number of training samples have better generalization properties that can well fit the entire sample space and work well on the testing samples.

In practical, disease diagnostic speed is an important index for the real-time and dynamical regulation of agricultural production. Here, we further carry out an experiment to evaluate the average online diagnosis time of each spectral curve sample achieved by different recognition methods with different enrollment size, and the results are shown in Table 7. It shows that ESRC has the highest time cost between about 2.5 to 6.5 ms, while that of the SVM ranks second and is between about 2.7 to 3 ms. The diagnostic time of LDA, ECR, and K-means is relatively small and between about 1 and 1.2 ms, which can meet the real-time requirements. Besides, it can be seen that the diagnostic time of the ECR and ESRC method slightly gets longer as the enrollment size increases; the reason may be that more training samples will increase the number of atoms in spectral libraries, causing the raise of the computation and time costs when solving the $l_{1}$-norm or $l_{2}$-norm regularized least squares problems. To sum up, the ECR method not only reaches the highest disease recognition accuracies but also has fast diagnostic speed, even if the number of training samples per type of disease is very small, demonstrating that the goal of reducing the required training samples and promoting the diagnosis accuracy is well achieved.

## 4. Conclusions

An ECR-based classification model is presented in this paper and we evaluate its performance by applying it to the cucumber disease recognition problem. For cucumber disease recognition, we first probe the refined spectral information related to disease using HSI technique, and then, construct pure and variation spectral libraries to respectively characterize the pure spectral curves and linear interferences introduced by illumination, occlusion or other factors. Given a query sample, it is collaboratively represented as a linear combination of all atoms from the spectral libraries and the coefficient vector is utilized to identify which disease it is infected with. A number of experiments are conducted to study the influences of preprocessing, sample dimension, variation spectral library, and enrollment size on the disease recognition effect of the ECR method. The results indicate that the ECR-based classification method could achieve high recognition accuracies and fast online diagnostic speed, even in the case of very few training samples, which could meet the needs of rapid and accurate non-destructive diagnosis to some extent in practical production.

## Figures and Tables

**Table 1:** A brief description of the groups.

Groups	Disease Type	Number of Plants	Number of Spectral Curves
A	Healthy	5	4000
B	Corynespora cassiicola	25	4000
C	Anthracnose	25	4000

**Table 2:** Parameter settings for different preprocessing methods.

Methods	Window Width	Polynomial Order	The Ideal Spectra
MAS	7	/	/
SG	7	3	/
MSC	/	/	The mean of all spectral curves

**Table 3:** Cucumber disease recognition accuracies under different preprocessing methods.

Methods	SG	MAS	SNV	MSC	SG-1st Der	SG-2nd Der
ESRC	92.08%	92.65%	69.99%	61.92%	82.94%	93.25%
SVM	92.95%	95.53%	82.61%	63.01%	90.46%	92.75%
LDA	89.02%	91.10%	70.12%	47.50%	82.36%	88.22%
K-means	93.74%	92.61%	73.90%	64.30%	90.82%	91.21%
ECR	95.48%	96.02%	63.70%	71.59%	89.37%	94.53%

**Table 4:** The results of ECR-based disease recognition method under different window widths.

**Window Widths**	3	5	7	9	11
**Disease Recognition Accuracies**	94%	95.7%	96%	95.7%	94.6%

**Table 5:** Disease recognition accuracies via different number of principal components.

Methods	Number of Principal Components									
	3	5	10	15	25	50	75	100	125	150
ESRC	87.7%	93.6%	93.2%	89.6%	93.9%	93.7%	93.4%	92.7%	94.0%	93.5%
SVM	94.3%	93.8%	91.9%	94.1%	95.0%	93.9%	56.9%	57.4%	56.4%	57.2%
LDA	83.9%	77.5%	81.8%	77.6%	90.1%	92.8%	93.3%	93.3%	89.1%	78.6%
K-means	93.4%	93.6%	93.7%	93.7%	93.7%	93.3%	93.6%	93.7%	93.8%	93.7%
RF	92.9%	94.9%	95.1%	95.6%	94.8%	94.7%	89.1%	92.1%	83.3%	88.6%
ECR	80.7%	95.8%	96.5%	96.7%	97.1%	96.2%	95.8%	94.7%	96.6%	96.6%

**Table 6:** Disease recognition accuracies when different enrollment size is adopted.

Methods	Enrollment Size *m* (the Number of Training Samples per Disease)								
	20	30	40	50	60	70	80	90	100
ESRC	94.5%	94.6%	95.4%	94.4%	95.5%	94.9%	94.8%	94.5%	94.9%
SVM	65.6%	70.3%	73.5%	81.3%	87.7%	93.2%	96.8%	96.8%	97.7%
LDA	70.9%	75.6%	76.4%	78.1%	80.4%	82.1%	83.4%	82.6%	83.8%
K-means	93.7%	93.7%	93.7%	93.6%	93.6%	93.6%	93.6%	93.7%	93.7%
ECR	97.6%	97.1%	98.1%	97.6%	97.4%	97.7%	98.3%	98.2%	98.5%

**Table 7:** The average online diagnostic time (ms) of each query sample with respect to different recognition methods.

Methods	Enrollment Size *m* (the Number of Training Samples per Disease)									
	10	20	30	40	50	60	70	80	90	100
ESRC	2.49	3.24	3.65	4.26	4.45	4.81	5.05	5.67	5.77	6.53
SVM	2.75	2.69	2.69	2.80	2.72	2.77	2.88	2.73	2.87	3.00
LDA	1.01	1.01	1.03	1.05	1.01	1.07	1.02	1.02	1.15	1.17
K-means	1.04	1.04	1.04	1.09	1.04	1.09	1.04	1.04	1.18	1.19
ECR	0.99	1.04	1.05	1.09	1.04	1.11	1.07	1.06	1.19	1.22
